# Gene expression changes and DNA damage after ex vivo exposure of peripheral blood cells to various CT photon spectra

**DOI:** 10.1038/s41598-021-91023-7

**Published:** 2021-06-08

**Authors:** Hanns Leonhard Kaatsch, Benjamin Valentin Becker, Simone Schüle, Patrick Ostheim, Kai Nestler, Julia Jakobi, Barbara Schäfer, Thomas Hantke, Marc A. Brockmann, Michael Abend, Stephan Waldeck, Matthias Port, Harry Scherthan, Reinhard Ullmann

**Affiliations:** 1 Bundeswehr Institute of Radiobiology Affiliated to Ulm University, Neuherbergstrasse 11, 80937 Munich, Germany; 2 Department of Radiology, Bundeswehr Central Hospital Koblenz, Rübenacher Straße 170, 56072 Koblenz, Germany; 3 Department of Neuroradiology, University Medical Center Mainz, Langenbeckstrasse 1, 55101 Mainz, Germany

**Keywords:** Gene expression, Genomics, Sequencing, Double-strand DNA breaks, High-throughput screening, Medical imaging, Tomography

## Abstract

Dual-energy CT provides enhanced diagnostic power with similar or even reduced radiation dose as compared to single-energy CT. Its principle is based on the distinct physical properties of low and high energetic photons, which, however, may also affect the biological effectiveness and hence the extent of CT-induced cellular damage. Therefore, a comparative analysis of biological effectiveness of dual- and single-energy CT scans with focus on early gene regulation and frequency of radiation-induced DNA double strand breaks (DSBs) was performed. Blood samples from three healthy individuals were irradiated ex vivo with single-energy (80 kV and 150 kV) and dual-energy tube voltages (80 kV/Sn150kV) employing a modern dual source CT scanner resulting in Volume Computed Tomography Dose Index (CTDIvol) of 15.79–18.26 mGy and dose length product (DLP) of 606.7–613.8 mGy*cm. Non-irradiated samples served as a control. Differential gene expression in peripheral blood mononuclear cells was analyzed 6 h after irradiation using whole transcriptome sequencing. DSB frequency was studied by 53BP1 + γH2AX co-immunostaining and microscopic evaluation of their focal accumulation at DSBs. Neither the analysis of gene expression nor DSB frequency provided any evidence for significantly increased biological effectiveness of dual-energy CT in comparison to samples irradiated with particular single-energy CT spectra. Relative to control, irradiated samples were characterized by a significantly higher rate of DSBs (p < 0.001) and the shared upregulation of five genes, *AEN*, *BAX*, *DDB2*, *FDXR* and *EDA2R*, which have already been suggested as radiation-induced biomarkers in previous studies. Despite steadily decreasing doses, CT diagnostics remain a genotoxic stressor with impact on gene regulation and DNA integrity. However, no evidence was found that varying X-ray spectra of CT impact the extent of cellular damage.

## Introduction

Dual-energy computed tomography (DECT) has become a well-established and widely used technique in modern medical diagnostics. Image acquisition at two energy levels—typically 70–80 kV and 140–150 kV—takes advantage of the different physical properties of low and high energetic photons to unravel the elemental composition of materials, which would not be resolvable based on CT numbers of voxels defined by single-energy CT (SECT) alone^1^. The combination of two attenuation measurements enhanced the power to delineate different tissues and paved the way for novel applications, e.g. in cardiovascular, thoracic, bone and abdominal imaging^2^.

Next to diagnostic benefits, DECT offers the chance of significant dose reduction, in particular by abolishing the obligation of a complete unenhanced scan due to reconstruction of virtual unenhanced images^3–7^. Nevertheless, comparative risk assessment of SECT and DECT should not only focus on radiation dose and dose rate, but also has to consider differences in biological effectiveness (BE) associated with varying photon energies^8–10^. In case of DECT, BE of the contributing energy spectra may presumably account only for a small fraction of individual risk in comparison to dose reduction opportunities by DECT in clinical routine. Yet, it might be a pivotal point of criticism as to risk assessment by means of the linear no-threshold model which is routinely applied in clinical risk assessment.

The importance of higher BE associated with exposure to low-energy photons has already been addressed in the context of mammography (X-ray tube voltage usually 25–35 kV). Several in vitro experiments comparing low-energy mammography X-rays with higher energetic radiation qualities showed an increase in dicentric chromosomes^11^, neoplastic cell transformation^12–15^, enhanced mutagenicity^16^ and micronuclei induction^17^. This raises the question whether the low-energy portion in X-ray spectra of CT might cause measurable differences in biological effectiveness between SE- und DECT which could reduce the apparent benefit of DECT. Against this background there is need for a profound knowledge-base that could assist radiologists in finding the optimal risk–benefit balance for DECT. To the best of our knowledge only one study so far has investigated biological effects of single- versus dual-energy scans and found no significant differences after pulmonary CT angiography comparing single- and dual-energy CT protocols based on the measurement of DNA double strand breaks (DSB) by means of the γH2AX-assay^18^.

In this study we aimed to gain further insights into the varying biological response possibly associated with very low dose, low-energy X-ray exposure during DECT. In the controlled setting of an ex vivo exposure of peripheral blood cells to adapted modern SECT and DECT scan protocols, we investigated differences in gene expression and the extent of DNA DSBs between the two CT techniques by means of whole transcriptome sequencing and 53BP1/γH2AX co-immunostaining^19^, respectively.

## Results

### Transcriptional response to irradiation with varying tube voltages

**Figure 1:**
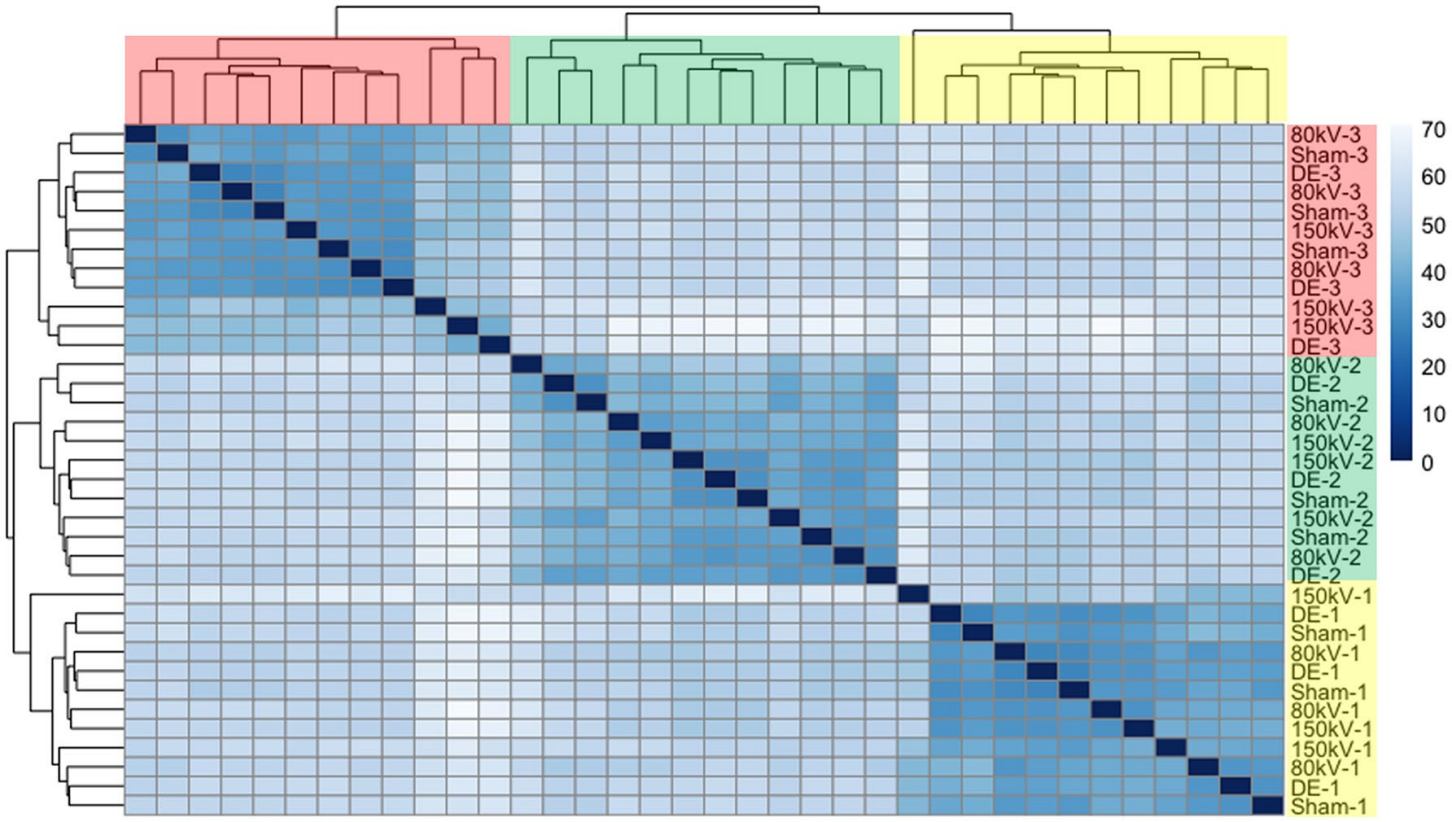
Unsupervised hierarchical clustering based on sample-to-sample distances of all 36 RNA sequencing experiments. Naming of samples indicates treatment (Sham, 80 kV, 150 kV and DE) and proband (1, 2, 3). Probands have additionally been highlighted in yellow (proband 1), green (proband 2) and red (proband 3) to the right and at the top of the heatmap. The order of samples in combination with the dendrogram at the top or to the left of the heatmap denote the clusters of samples arising as a result of the hierarchical clustering process. Given the dominance of inter-individual differences of gene expression profiles over CT-induced effects, all samples derived from the respective probands group together in distinct clusters, independent of whether they have been irradiated or not. Within the heatmap sample-to-sample distance based on log2-transformed normalized read count data is displayed by a blue color gradient with dark blue indicating a high degree of similarity.

**Figure 2:**
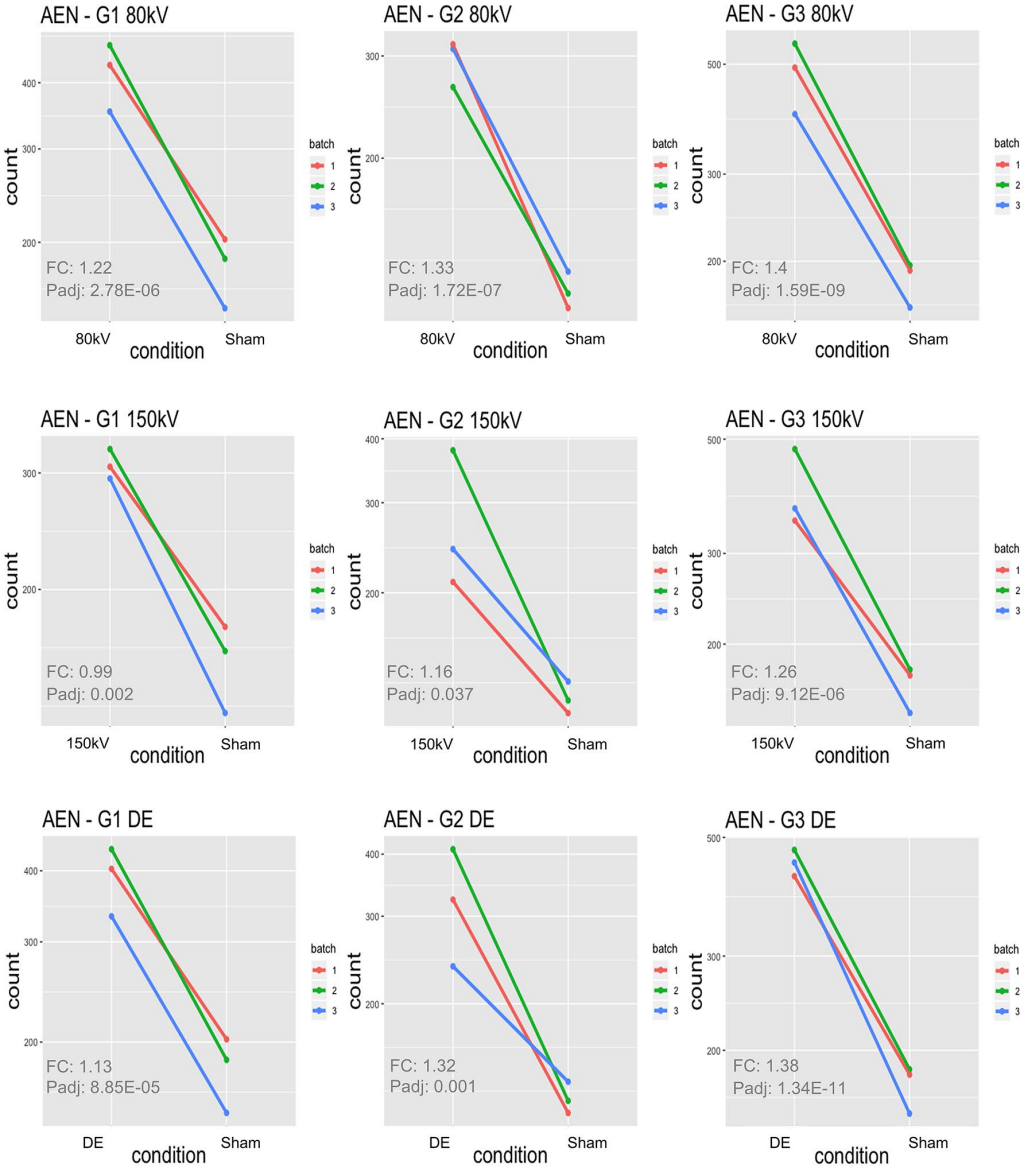
Gene expression plots for *AEN* for 80 kV single-energy CT (SECT), 150 kV SECT and dual-energy CT (DE) irradiation: Each plot depicts gene counts obtained from RNA sequencing data of the respective proband (G1-3) with post-irradiation gene count displayed to the left and corresponding sham-irradiated gene counts displayed to the right. Colored lines (green, blue and red) indicate the three replicate experiments.

The overall effect of CT exposure on gene expression was only moderate. Unsupervised hierarchical clustering based on sample-to-sample distances indicated the clear dominance of inter-individual differences over irradiation associated effects. As depicted in Fig. 1 all samples of each individual clustered together, irrespective of whether they had been irradiated or not. In line with this observation, only few genes were found deregulated with respect to corresponding sham irradiated controls (18–109 for 80 kV, 9–67 for 150 kV and 10–21 for dual-energy acquisition setting; Supplementary Table S1). Among the significantly deregulated genes, *AEN* (*Apoptosis Enhancing Nuclease*) was shared between all replicates, probands and CT protocols (Fig. 2).

**Figure 3:**
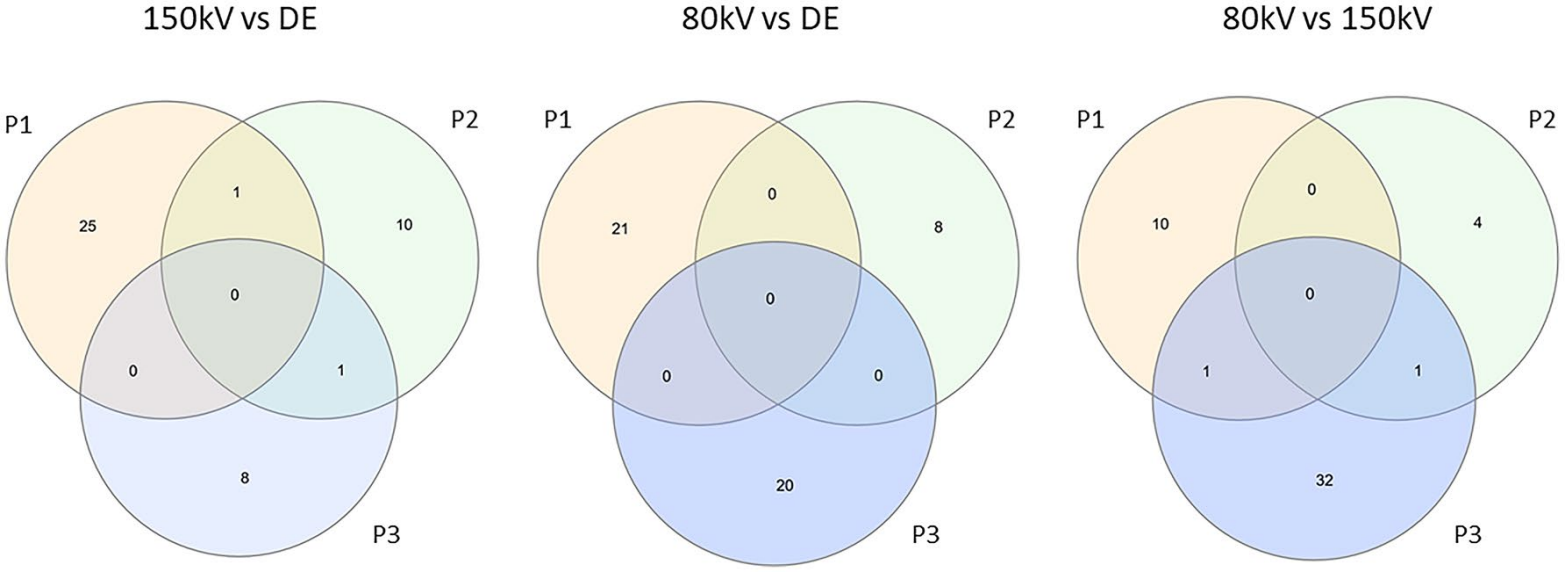
Venn diagram summarizing the number of differentially expressed genes identified by direct comparison of 80 kV single-energy CT (SECT), 150 kV SECT and dual-energy CT (DE), respectively. The corresponding gene lists can be found in Supplementary Table S2.

A direct comparison of 80 kV, 150 kV and DECT experiments did not reveal any common gene expression signature characteristic for one of the three tube voltages. In total this direct comparison identified 48, 49 and 45 significantly differentially expressed genes. None of them was shared among all probands (Fig. 3).

**Figure 4:**
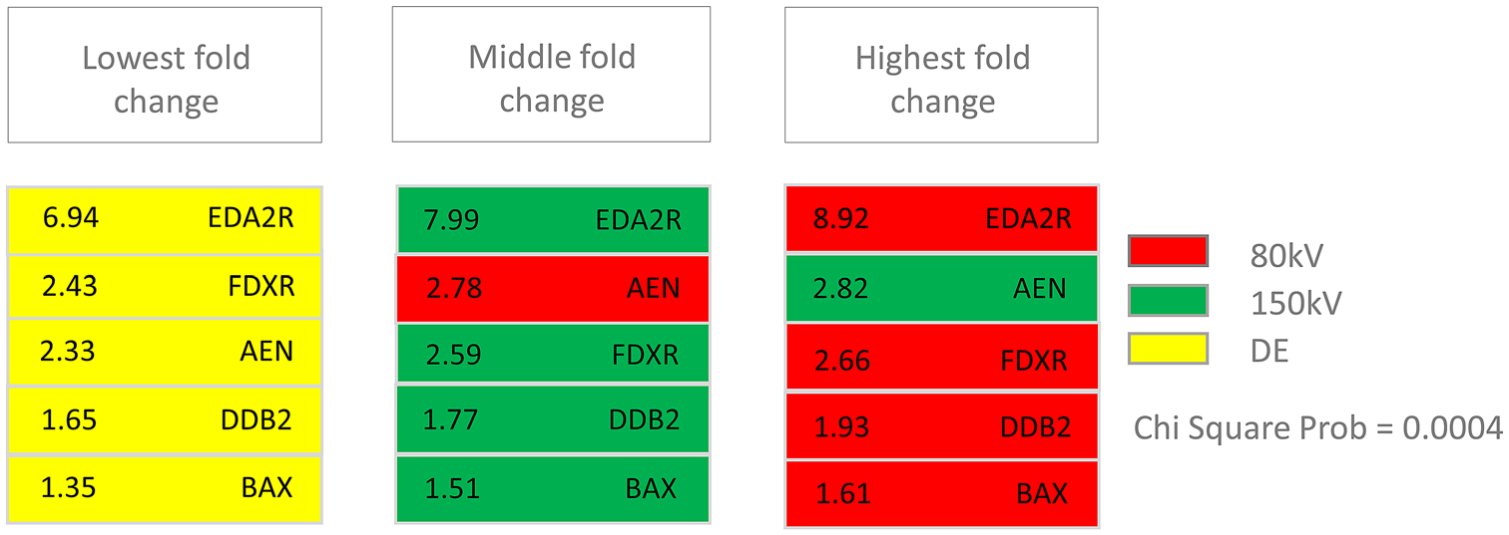
Frequency distribution of mean fold changes on a linear scale for the genes *EDA2R*, *FDXR*, *AEN*, *DDB2* and *BAX*. Mean fold changes for each gene and treatment averaged over all probands and replicates were categorized according to whether they represent the lowest, middle or highest fold change of each gene. Exposure groups are indicated by red (80 kV), green (150 kV) and yellow (dual-energy (DE)) coloring of boxes. Mean fold changes given in the boxes refer to qRT-PCR results based on nine measurements (3 probands × 3 technical replicates per proband). See “Results” section for details.

A second round of analysis with less stringent criteria (see “Materials and methods”) added *BAX*, *DDB2*, *EDA2R* and *FDXR* to the list of CT associated candidate genes. Upregulation relative to corresponding sham irradiated controls was verified for all of these five CT associated genes by qRT-PCR (Supplementary Fig. S1). As the good concordance of sequencing and qRT-PCR data suggested a high accuracy of fold-change quantification, we further investigated the dependency of fold-change magnitude on the type of CT exposure. For this purpose we calculated the average fold-change for each gene and treatment (i.e. the average over three individuals and their three replicates). The resulting 15 average fold changes (five genes x three exposure types) were categorized according to whether they represent the lowest, middle or highest fold-change of each gene. The frequency distribution within these categories was non-random (Chi square p = 0.0004), with all fold-changes induced by DECT falling in the lowest fold-change category. Apart from *AEN,* highest fold-changes for each gene were associated with 80 kV SECT, whereas four out of five middle fold-changes were induced by 150 kV SECT (Fig. 4). The same trend was found when considering probands individually (Chi Square p = 0.0342).

Within the sets of up-regulated genes, functional enrichment analysis identified shared overrepresentation of transcriptional regulatory processes (p ≤ 1.32 × 10^−4^) in either single-energy level of two probands and of intrinsic apoptotic signaling pathways (p ≤ 5.95 × 10^–5^) partly linked to the WNT signaling pathway (p ≤ 4.16 × 10^−5^) in 80 kV and dual-energy setting of two probands.

### DNA double strand break frequency after SE- and DECT exposure

**Figure 5:**
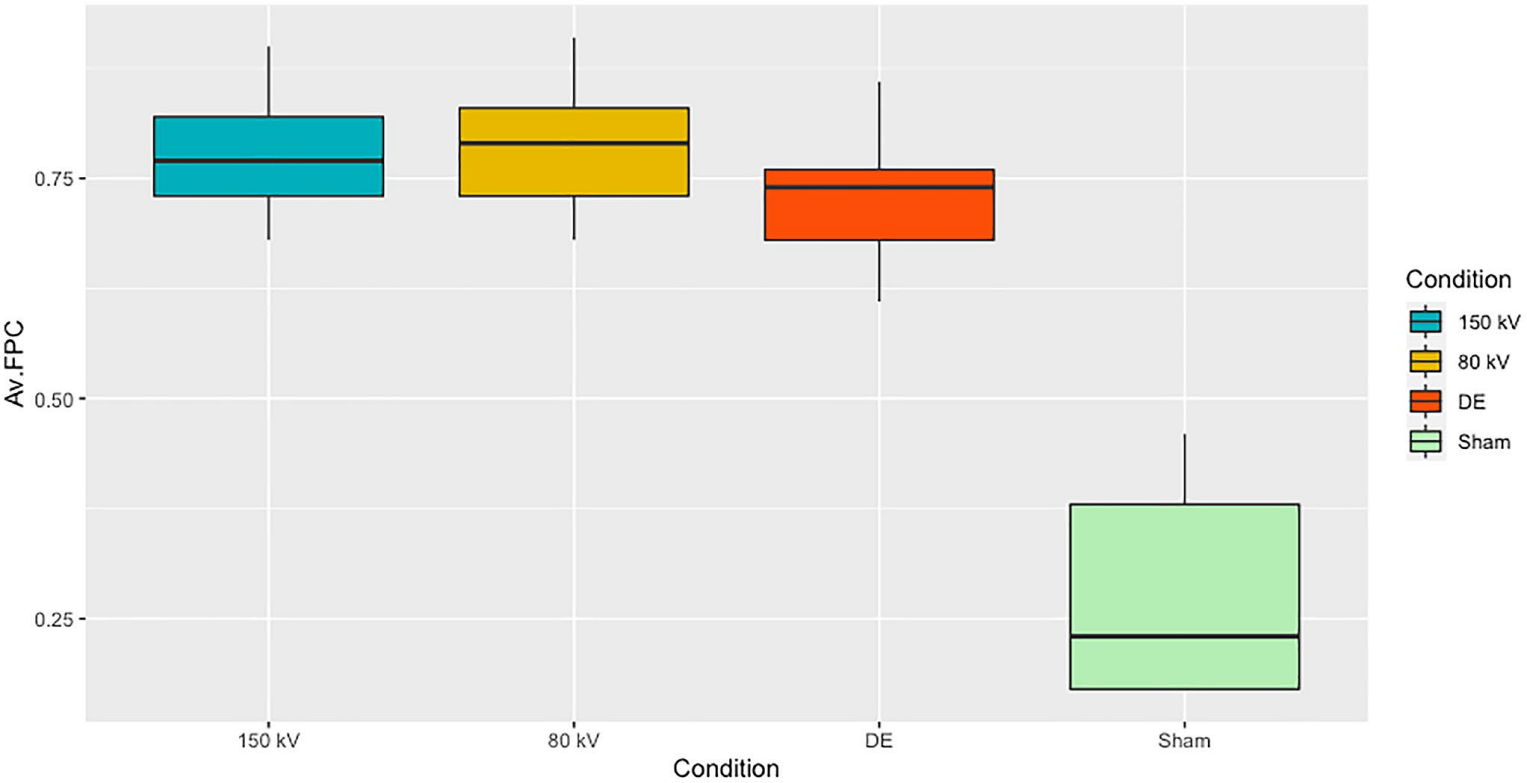
Average number of DNA double strand break (DSB) foci observed without (Sham) and after 80 kV, 150 kV and dual-energy CT (DE) irradiation. Each box represents the interquartile range (25th to 75th percentile) of average DSBs per cell observed in three probands and their three replicates. Whiskers indicate the minimum and maximum. The median is shown as horizontal line within the boxes. Relative to control all CT applications led to a highly significant increase in DSB (p < 0.001). Relative to SECT the average number of foci per cell appears slightly lower in samples exposed to DECT, but this difference did not reach significance (p > 0.15).

DNA double strand breaks (DSBs) were estimated by counting γ-H2AX + 53BP1 colocalizing foci in PBMCs^19,20^ of three different probands in triplicates (Fig. 5). The mean of the average number of baseline DSB foci per cell (FPC ± SD) detected in the control samples was 0.27 ± 0.11. For all three radiation modalities the CT-induced increase in average DSB FPC values over that of the sham irradiated samples was highly significant (p < 0.001). The average FPC values between the three irradiation modalities was 0.78 ± 0.077 for 80 kV, 0.78 ± 0.069 for 150 kV and 0.73 ± 0.073 for DECT, an insignificant difference (p > 0.15) between DECT and each of the SECT modalities. These data show that there is no difference for the average DSB numbers induced between the two single-energy CT modalities (p = 1) and between these two and the DE mode (p > 0.15). Hence, the different CT energies alone or in combination do not induce different amounts of DSBs indicating a similar biological effect of the different CT settings.

Scrutinizing the frequency distribution of DSBs analogous to the procedure described in the context of gene expression fold changes did not reveal any significant dependency of DSB frequency on the type of CT exposure.

## Discussion

Low energy photons emitted during DECT may inherit the risk of increased biological effectiveness, a fact that has to be taken into account when weighing up benefits and potential risks of DECT diagnostics. In this study, we investigated the consequences of ex vivo exposure of peripheral blood inside a X-ray phantom to 80 kV SECT, 150 kV SECT and DECT irradiation with regard to two biological endpoints: gene expression and DNA double strand break frequency.

Irrespective of the CT protocol applied, CT-associated effects on gene regulation were only moderate when compared to inter-individual differences of gene expression profiles. Nevertheless, we observed shared upregulation of five genes in CT-irradiated samples, namely, *AEN*, *DDB2*, *FDXR, BAX* and *EDA2R.* These five genes have already emerged as potential radiation biomarkers in studies with exposures to doses ≥ 50 mGy^21^ or ≥ 0.5 Gy^22,23^. Moreover, they largely overlap with the candidate genes defined in our previous CT study using 150 kV SECT alone^24^. Altogether, these findings underscore the potential of these genes as biomarkers for irradiation, whose upregulation can even be triggered by very low doses. Noteworthy, all of these five genes are known targets of the p53 tumor suppressor with impact on cell cycle control, apoptosis and DNA damage repair, activated by cellular damage^25–27^.

While we have found a common set of genes distinguishing irradiated and non-irradiated samples, it was not possible to further specify whether a sample was irradiated with 80 kV SECT, 150 kV SECT or DECT. This suggests that in the experimental settings used in this study, involving exposition to very low X-ray doses in conjunction with varying X-ray spectra, the fraction of low energy photons emitted during DECT diagnostics has no detectable impact on gene regulation. Yet, further studies are needed to verify that this statement holds true for other points of time post irradiation, higher dose values and in vivo studies. One limitation especially in the context of the low doses applied is that it cannot be ruled out that the polyenergetic spectra of X-ray irradiation or the use of a phantom may have masked subtle effects. Unfortunately, this uncertainty cannot be addressed in the current study as local dose and its variability is unknown and diverging spectra may affect the ratio of local to effective dose. Moreover, CTDIvol and DLP values may vary by a systematic deviation of ± 10% as specified by the manufacturer (System owner manual SOMATOM Force; Chapter 1.1.8 “Accuracy of Dose Indications”) along with additional differences depending on the applied spectrum.

Results at the level of gene regulation were mirrored by the outcome of DNA damage assessment based on the focal colocalization of 53BP1 and γ-H2AX DSB damage markers. Estimation of DSB frequency by means of these two dsDNA damage markers is a well-established approach^19,28,29^. In line with previous in vivo studies^18,20^ we clearly observed the induction of DSBs by CT irradiation in significant excess over base line rate (p < 0.001), allowing the clear separation of irradiated and non-irradiated samples, which agrees with other SECT DSB studies^20,30–32^. With regard to single- and dual-energy CT irradiation modalities, the DSBs numbers induced were similar to each other (Fig. 4). These results show that SECT or DECT elicit a similar amount of dsDNA damage, suggesting a comparable biological effect for this endpoint. Future studies of other endpoints like e.g., chromosome aberrations or micronuclei^33^ shall provide further insights into the radio-response of PBMCs to SECT or DECT.

Clearly, most of the potential limitations mentioned in the context of gene expression may also be valid for DNA double strand break analysis. However, unlike for gene expression the optimal time slot for DSB analysis is predefined by DNA repair kinetics, mitigating this matter of concern.

Altogether, the analysis of both gene regulation and DNA DSBs does not provide evidence for an increased biological effect of DECT as compared to 80 kV and 150 kV SECT. On the contrary, in light of the observation that the extent of upregulation of all genes tested by qRT-PCR is lower in DECT relative to 150 kV SECT and in particular to 80 kV SECT one may speculate that e.g. the modulation of radiation quality by the use of tin filtration, routinely added to the high-kilovolt tube during DECT exposures, could result in reduced biological effectiveness. Yet, given the rather small differences in gene upregulation observed in this study, this aspect has to be systematically addressed by future studies.

In conclusion, the results of this study do not suggest any increased biological effectiveness associated with the low energy spectrum of DECT. Yet, despite decreasing dose burden caused by CT scans, the genotoxic effect of CT irradiation still subsists and should remain a crucial aspect of risk assessment before performing CT diagnostics.

## Materials and methods

### Ex vivo CT exposure and dose estimation

Ethical approval for this study was obtained from the responsible ethical review committee of the Medical Association of Rhineland Palatinate, Germany (reference number: 837.084.17(10918)). All experiments were performed in accordance with relevant named guidelines and regulations. Written informed consent was obtained from all probands. Peripheral blood samples were collected from three healthy male donors (age 30, 32 and 53 years) in EDTA-Monovettes (5 ml; Sarstedt, Nümbrecht, Germany) in triplicates with matching sham control samples. Both, single-energy CT and dual-energy CT were performed utilizing adapted lumbar spine protocols in a 384 (2 × 192)—channel multi-detector row dual source CT scanner (SOMATOM Force, Siemens Medical Solutions, Forchheim, Germany) with blood samples placed in a lumbar spine phantom at room temperature. Acquisition parameters were set to X-ray tube voltages of 80 kV and 150 kV single source single-energy, respectively, and dual source dual-energy (80 kV/150 kV (with Sn filter)). To obtain identical dose values the current was manually set to 950 and 160 mA in SECT and 440 and 220 mA in DECT for 80 kV and 150 kV, respectively. Pitch was 0.6 and rotation time was 0.5 s at a scanning length of 367 mm for all protocols. The applied setting resulted in a Volume CT Dose Index (CTDIvol) of 15.79–18.26 mGy, in a dose length product (DLP) of 606.7–613.8 mGy*cm and in an administered effective dose of 7.0 ± 0.08 mSv as calculated for better context in accordance to ICRP 103 report by means of Monte Carlo model-based dose estimation implemented in ImpactDose (CT Imaging GmbH, Erlangen, Germany). After incubation for 6 h at 37 °C, a point of time chosen based on the results of our previous study^24^, samples were transferred into PAXgene Blood RNA tubes (PreAnalytiX, Hombrechtikon, Switzerland). The fraction of peripheral blood dedicated to DNA double strand break analysis was incubated for 20 min at 37 °C before lymphocytes were isolated by density centrifugation as detailed below.

### Gene expression analysis

Total RNA was isolated using the PAXgene Blood RNA Kit (PreAnalytiX, Hombrechtikon, Switzerland). Agilent 2100 Bioanalyzer System (Agilent, Santa Clara, CA, USA) was used for RNA quantification and quality assessment. RNA-Seq library preparation was conducted for all samples with RNA Integrity Numbers (RIN) equal or above 7 employing the NEBNext Ultra II Directional RNA Library Kit for Illumina following the manufacturers recommendations (NEBNext Ultra™ II Directional RNA Library Prep Kit for Illumina and rRNA depletion option, New England Biolabs Inc., Ipswich, MA, USA, protocol version 1.0 4/17). After quantification using a Qubit 3.0 Fluorometer (Thermo Scientific, Waltham, MA, USA) and quality control by means of Agilent Bioanalyzer system (Agilent 2100 Bioanalyzer, Agilent, Santa Clara, CA, USA), samples were sequenced on the Illumina NextSeq500 sequencing platform (single-end; 1 × 75 bp; high output reagent kit).

Differential gene expression analysis was based on a workflow suggested by Love et al.^34^. In brief, read mapping to the human reference transcriptome (GRCh37.67), quantifying transcript abundance and correcting for fragment-level gc-biases was performed with Salmon (version 0.8.1)^35^. Salmon results were imported via *tximport* (version 1.4.0)^36^ into DESeq2 (version 1.22.2)^34^. Overall estimation of similarities and differences between treatments was done by means of unsupervised hierarchical clustering of sample-to-sample distances based on log2 transformed normalized read counts using the default settings of DESeq2. The hierarchical clustering algorithm groups samples based on the similarities of their gene expression profiles and visualizes this clustering in the form of a dendrogram. The number of clusters is not predefined (unsupervised), but arises as a result of sample composition. To identify the number of genes that were commonly deregulated between the different exposure conditions, differentially expressed genes were displayed using a Venn diagram made with InteractiVenn^37^. Significant differential gene expression was defined by a log2 fold change of FPKM (fragments per kilobase per million mapped fragments) >|0.5| and P_adj_ (FDR-adjusted p-value) < 0.1. This stringent analysis was complemented by an additional search for candidate genes using slackened criteria in order to accommodate to the subtle effects that can be expected in light of the very low doses applied during CT scans, i.e. a log2 fold change of FPKM >|0.5| and an uncorrected p value < 0.05 in at least two of the three probands. All candidate genes fulfilling these criteria were tested by real-time quantitative polymerase chain reaction (qRT-PCR) in all replicates of all probands.

AmiGO 2^38,39^ was used for gene enrichment analysis applying Fisher’s exact test in combination with False Discovery Rate (FDR p < 0.05) correction ^40,41^.

### DNA double strand break analysis by means of γ-H2AX + 53BP1 co-immunostaining

The frequency of DNA DSBs within peripheral blood mononuclear cells (PBMC) was determined by microscopic quantification of focal accumulations of γ-H2AX and 53BP1^19^, two marker proteins indicative for the presence of a DSB. For this purpose, PBMCs were isolated from blood samples by Ficoll Paque Plus (Merck, Darmstadt, Germany) density centrifugation. PBMCs were washed twice with PBS, fixed in 70% ethanol and stored at − 20 °C. Immunofluorescence staining and DSB foci analysis was performed as described previously^19,20,42^. Using a Zeiss Axioimager Z2 epifluorescence microscope of the MetaSystems (Altlussheim, GER) ISIS imaging system equipped with appropriate single and dual-band filter sets co-localizing γ-H2AX + 53BP1 foci were counted in 100 well separated and morphologically intact cells by an experienced investigator (HS).

### RNA-Seq validation using real-time polymerase chain reaction (qRT-PCR)

Aliquots of total RNA (1 µg) were reverse transcribed with the High-Capacity cDNA Reverse Transcription Kit (Applied Biosystems, Life Technologies, Darmstadt, Germany). PCR reactions were performed in a 96-well format using commercial TaqMan assays (*AEN*, Hs00224322_m1; *DDB2*, Hs00172068_m1; *FDXR*, Hs01031617_m1; *BAX*, Hs00180269_m1; *EDA2R*, Hs00939736_m1; *18S* rRNA, Hs03003631_g1) following the QuantStudio 12 K Flex Real-Time PCR System amplification protocol. Cycle threshold (Ct) values of the five genes were normalized relative to *18S* rRNA. The ratio/fold-change (FC) relative to the unexposed sample at the same time point was determined. A FC of one corresponds to a gene expression similar to unexposed samples. A FC higher or lower than one refers to a several-fold over- or under-expression of the gene of interest after exposure relative to the reference. All materials and consumables used were ordered from ThermoFisher/Applied Biosystems (Weiterstadt, Germany).

## Supplementary Information

Supplementary Table 1.

Supplementary Table 2.

Supplementary Table 3.

Supplementary Figure 1.

Supplementary Figure 2.

## Data Availability

Data access is available through GEO Series accession number GSE156187 (https://www.ncbi.nlm.nih.gov/geo/query/acc.cgi?acc=GSE156187) by deposition in NCBI’s Gene Expression Omnibus^40,41^.

## References

[1] McCollough CH (2020). Principles and applications of multienergy CT: Report of AAPM task group 291. Med. Phys..

[2] Goo HW, Goo JM (2017). Dual-energy CT: New horizon in medical imaging. Korean J. Radiol..

[3] Takeuchi M (2012). Split-bolus CT-urography using dual-energy CT: feasibility, image quality and dose reduction. Eur. J. Radiol..

[4] Flors L, Leiva-Salinas C, Norton PT, Patrie JT, Hagspiel KD (2013). Endoleak detection after endovascular repair of thoracic aortic aneurysm using dual-source dual-energy CT: Suitable scanning protocols and potential radiation dose reduction. AJR Am. J. Roentgenol..

[5] Buffa V (2014). Dual-source dual-energy CT: dose reduction after endovascular abdominal aortic aneurysm repair. Radiol. Med. (Torino).

[6] Chen CY (2015). Split-bolus portal venous phase dual-energy CT urography: Protocol design, image quality, and dose reduction. AJR Am. J. Roentgenol..

[7] Pulickal GG, Singh D, Lohan R, Chawla A (2019). Dual-source dual-energy CT in submandibular sialolithiasis: Reliability and radiation burden. AJR Am. J. Roentgenol..

[8] Paget V (2019). Multiparametric radiobiological assays show that variation of X-ray energy strongly impacts relative biological effectiveness: Comparison between 220 kV and 4 MV. Sci. Rep..

[9] Hill MA (2004). The variation in biological effectiveness of X-rays and gamma rays with energy. Radiat. Prot. Dosim..

[10] Hunter N, Muirhead CR (2009). Review of relative biological effectiveness dependence on linear energy transfer for low-LET radiations. J. Radiol. Prot..

[11] Schmid E, Regulla D, Kramer H-M, Harder D (2002). The effect of 29 kV X rays on the dose response of chromosome aberrations in human lymphocytes. Radiat. Res..

[12] Frankenberg D, Kelnhofer K, Bär K, Frankenberg-Schwager M (2002). Enhanced neoplastic transformation by mammography X Rays relative to 200 kVp X Rays: Indication for a strong dependence on photon energy of the RBEM for various end points. Radiat. Res..

[13] Göggelmann W (2003). Re-evaluation of the RBE of 29 kV x-rays (mammography x-rays) relative to 220 kV x-rays using neoplastic transformation of human CGL1-hybrid cells. Radiat. Environ. Biophys..

[14] Heyes GJ, Mill AJ (2004). The neoplastic transformation potential of mammography X rays and atomic bomb spectrum radiation. Radiat. Res..

[15] Brenner DJ, Amols HI (1989). Enhanced risk from low-energy screen–film mammography X rays. Br. J. Radiol..

[16] Frankenberg-Schwager M (2002). Mutagenicity of low-filtered 30 kVp X-rays, mammography X-rays and conventional X-rays in cultured mammalian cells. Int. J. Radiat. Biol..

[17] Słonina D (2003). Induction of micronuclei in human fibroblasts and keratinocytes by 25 kV x-rays. Radiat. Environ. Biophys..

[18] Tao SM (2018). Comparison of the effect of radiation exposure from dual-energy CT versus single-energy CT on double-strand breaks at CT pulmonary angiography. Eur. J. Radiol..

[19] Eberlein U, Peper M, Fernández M, Lassmann M, Scherthan H (2015). Calibration of the γ-H2AX DNA double strand break focus assay for internal radiation exposure of blood lymphocytes. PLoS ONE.

[20] Schumann S (2020). DNA damage in blood leukocytes of prostate cancer patients undergoing PET/CT examinations with [(68)Ga]Ga-PSMA I&T. Cancers.

[21] Sokolov M, Neumann R (2016). Global gene expression alterations as a crucial constituent of human cell response to low doses of ionizing radiation exposure. Int. J. Mol. Sci..

[22] Paul S, Amundson SA (2008). Development of gene expression signatures for practical radiation biodosimetry. Int. J. Radiat. Oncol. Biol. Phys..

[23] Zheng J (2015). Gene expression profiling in non-human primate jejunum, ileum and colon after total-body irradiation: A comparative study of segment-specific molecular and cellular responses. BMC Genom..

[24] Kaatsch HL (2020). CT irradiation-induced changes of gene expression within peripheral blood cells. Health Phys..

[25] Allen MA (2014). Global analysis of p53-regulated transcription identifies its direct targets and unexpected regulatory mechanisms. Elife.

[26] Jeay S (2015). A distinct p53 target gene set predicts for response to the selective p53-HDM2 inhibitor NVP-CGM097. Elife.

[27] Fischer M (2017). Census and evaluation of p53 target genes. Oncogene.

[28] Marková E, Schultz N, Belyaev IY (2007). Kinetics and dose-response of residual 53BP1/gamma-H2AX foci: Co-localization, relationship with DSB repair and clonogenic survival. Int. J. Radiat. Biol..

[29] Rothkamm K (2015). DNA damage foci: Meaning and significance. Environ. Mol. Mutagen.

[30] Jafarpour SM (2018). Evaluation of ameliorative potential of vitamins E and C on DNA double strand break (DSB) in patients undergoing computed tomography (CT): A clinical study. ijmcmed.

[31] Halm BM (2014). γ-H2AX foci are increased in lymphocytes in vivo in young children 1 h after very low-dose X-irradiation: A pilot study. Pediatr. Radiol..

[32] Vandevoorde C (2015). EPI-CT: in vitro assessment of the applicability of the gamma-H2AX-foci assay as cellular biomarker for exposure in a multicentre study of children in diagnostic radiology. Int. J. Radiat. Biol..

[33] da Fonte JB, Andrade TM, Albuquerque RL, de Melo MFB, Takeshita WM (2018). Evidence of genotoxicity and cytotoxicity of X-rays in the oral mucosa epithelium of adults subjected to cone beam CT. Dentomaxillofac Radiol..

[34] Love MI, Huber W, Anders S (2014). Moderated estimation of fold change and dispersion for RNA-seq data with DESeq2. Genome Biol..

[35] Patro R, Duggal G, Love MI, Irizarry RA, Kingsford C (2017). Salmon provides fast and bias-aware quantification of transcript expression. Nat. Methods.

[36] Soneson C, Love MI, Robinson MD (2016). Differential analyses for RNA-seq: transcript-level estimates improve gene-level inferences. F1000Research.

[37] Heberle H, Meirelles GV, da Silva FR, Telles GP, Minghim R (2015). InteractiVenn: A web-based tool for the analysis of sets through Venn diagrams. BMC Bioinform..

[38] Ashburner M (2000). Gene ontology: Tool for the unification of biology. Nat. Genet..

[39] Mi H, Muruganujan A, Ebert D, Huang X, Thomas PD (2019). PANTHER version 14: More genomes, a new PANTHER GO-slim and improvements in enrichment analysis tools. Nucleic Acids Res..

[40] Barrett T (2013). NCBI GEO: Archive for functional genomics data sets–update. Nucleic Acids Res..

[41] Edgar R, Domrachev M, Lash AE (2002). Gene Expression Omnibus: NCBI gene expression and hybridization array data repository. Nucleic Acids Res..

[42] Lamkowski A (2014). DNA damage focus analysis in blood samples of minipigs reveals acute partial body irradiation. PLoS ONE.

