# Supplementary figures and images for: Gene expression changes and DNA damage after ex vivo exposure of peripheral blood cells to various CT photon spectra
Source: Sci Rep. 2021 Jun 8;11:12060. doi: 10.1038/s41598-021-91023-7 (PMC8187728; doi:10.1038/s41598-021-91023-7)

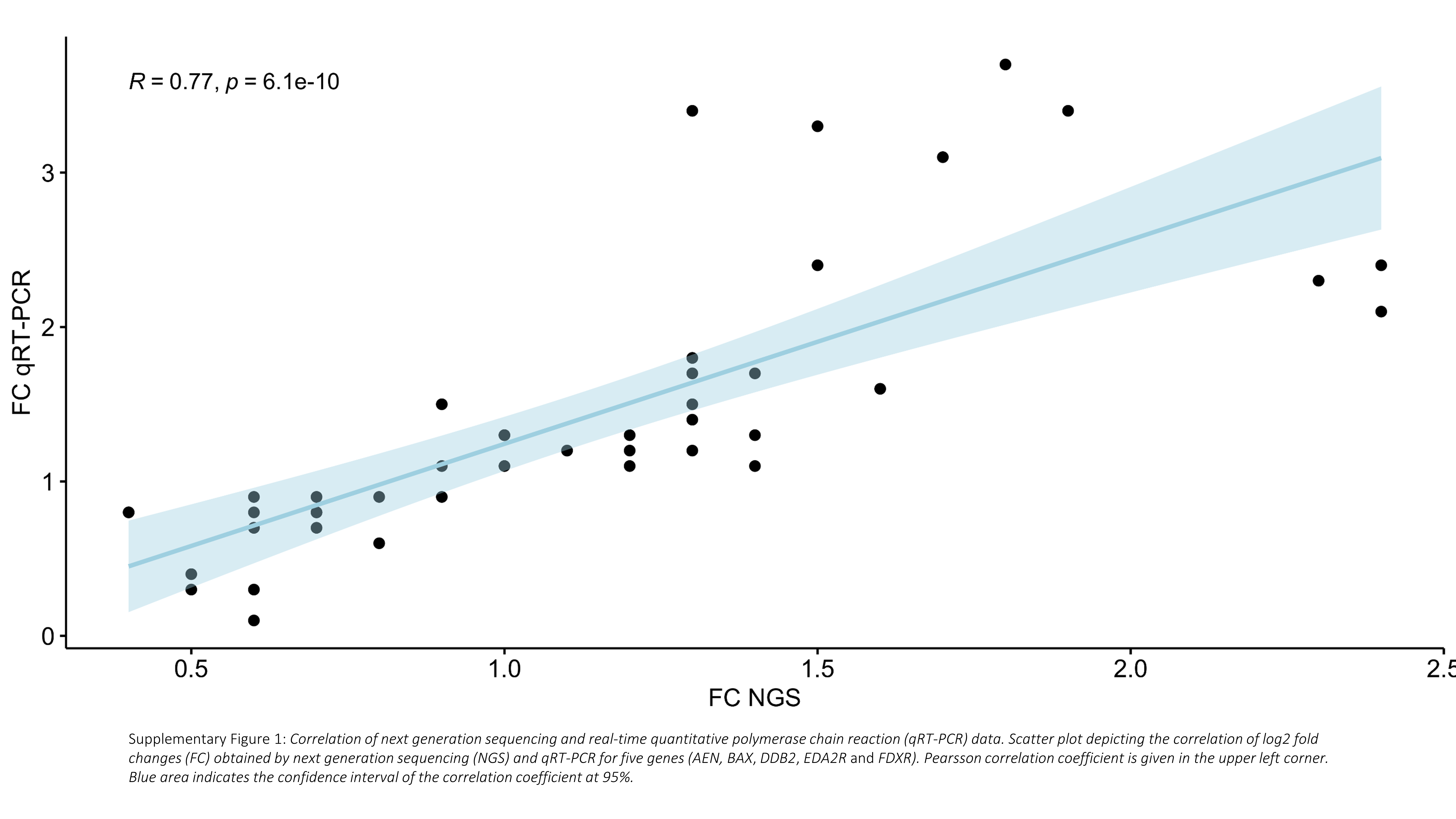

Supplement: Supplementary file 4 — Supplementary Figure 1. [file 41598_2021_91023_MOESM4_ESM.tif]

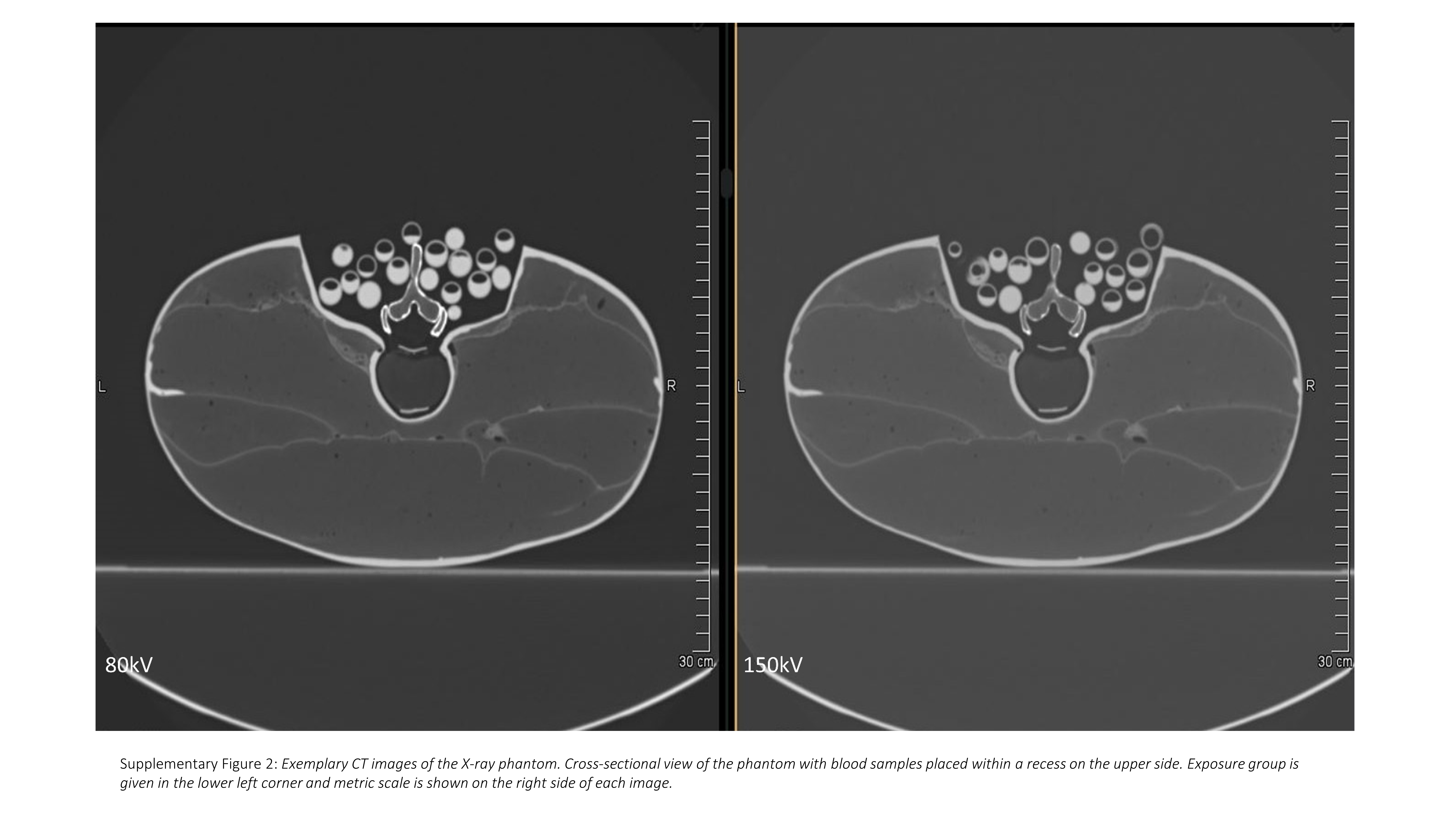

Supplement: Supplementary file 5 — Supplementary Figure 2. [file 41598_2021_91023_MOESM5_ESM.tif]
